# Omega-3 Fatty Acids and Insulin Resistance: Focus on the Regulation of Mitochondria and Endoplasmic Reticulum Stress

**DOI:** 10.3390/nu10030350

**Published:** 2018-03-14

**Authors:** Marilena Lepretti, Stefania Martucciello, Mario Alberto Burgos Aceves, Rosalba Putti, Lillà Lionetti

**Affiliations:** 1 Department of Chemistry and Biology, University of Salerno, Via Giovanni Paolo II, 132, Fisciano 84084, Italy; mlepretti@unisa.it (M.L.); smartucciello@unisa.it (S.M.); mburgosaceves@unisa.it (M.A.B.A.); 2 Department of Biology, University of Naples Federico II, Complesso Universitario di Monte S.Angelo, Edificio 7, via Cintia 26, 80126 Napoli, Italy; rosalba.putti@unina.it

**Keywords:** mitochondrial dysfunction, mitofusin, inflammasome, MAM, EPA, DHA, fish oil, oxidative stress

## Abstract

Mitochondrial dysfunction and endoplasmic reticulum (ER) stress have been suggested to play a key role in insulin resistance development. Reactive oxygen species (ROS) production and lipid accumulation due to mitochondrial dysfunction seemed to be important mechanisms leading to cellular insulin resistance. Moreover, mitochondria are functionally and structurally linked to ER, which undergoes stress in conditions of chronic overnutrition, activating the unfolded protein response, which in turn activates the principal inflammatory pathways that impair insulin action. Among the nutrients, dietary fats are believed to play key roles in insulin resistance onset. However, not all dietary fats exert the same effects on cellular energy metabolism. Dietary omega 3 polyunsaturated fatty acids (PUFA) have been suggested to counteract insulin resistance development by modulating mitochondrial bioenergetics and ER stress. In the current review, we summarized current knowledge on the role played by mitochondrial and ER stress in inflammation and insulin resistance onset, focusing on the modulation role of omega 3 PUFA on these stress pathways. Understanding the mechanisms by which omega 3 PUFA modulates cellular metabolism and insulin resistance in peripheral tissues may provide additional details on the potential impact of omega 3 PUFA on metabolic function and the management of insulin resistance in humans.

## 1. Introduction

Insulin resistance is a condition in which the response of peripheral tissue to insulin is attenuated and precedes by several years the development of type 2 diabetes mellitus. The functional consequence of insulin resistance in various tissues has been reviewed by Martin and McGee [1]. Insulin resistance in the skeletal muscle and the liver has negative effects on glucose homeostasis. Skeletal muscle seems to play a significant role in whole body insulin resistance [2,3]. It accounts for ~80% of post-prandial glucose disposal. In conditions of insulin resistance, the impaired glucose uptake—primarily due to the defective regulation of glucose transporter isoform 4 (GLUT4)—has significant effects on whole body glucose homeostasis and insulin action [1]. In the liver, suppression of glucose output is impaired in the insulin resistant state, due to impaired suppression of gluconeogenesis and glycogenolysis [4]. In the white adipose tissue (WAT), the impaired suppression of lipolysis contributes to the hyperlipidemia seen in insulin resistant states [5]. In addition, the alteration in the secreted adipokine profile induced by insulin resistance leads to a pro-inflammatory state, which in turn has detrimental effects on other metabolic tissues [6]. Brown adipose tissue (BAT) also plays an important role in glucose homeostasis [7]. It is well known that BAT regulates whole-body energy homeostasis by dissipating chemical energy as heat via high levels of mitochondrial uncoupling protein 1. Several studies have also shown that BAT activity improves glucose homeostasis and clears about 75% of the total glucose from the circulation in rodents [7,8]. In adult humans, some studies suggested that cold exposure significantly increases glucose disposal in BAT [9,10] and obese individuals seem to exhibit a less pronounced response [11], supporting the notion that BAT may function as an antidiabetic tissue in humans [12].

The study of the cellular and molecular causes of insulin resistance is an important research area for strategies to prevent metabolic disorders that are responsible for the onset of type 2 diabetes mellitus and associated co-morbidities. Research efforts have been dedicated to the understanding of molecular mechanisms that cause insulin resistance onset and suggest that multifactorial and complex interactions are involved. 

At a mechanistic level, chronic low grade systemic inflammation and ectopic lipid accumulation in non-adipose tissues have been implicated in the development of insulin resistance [13]. Indeed, the “lipotoxicity theory” suggests that cytosolic ectopic accumulation of fatty acid metabolites, such as diacylglycerols (DAG) and/or ceramides, underlies insulin resistance development in peripheral metabolic tissues—namely skeletal muscle and liver (reviewed in Lark et al. [3]). On the other hand, the “inflammatory theory” suggested a link between elevated systemic and tissue inflammation with insulin resistance [3,14]. High fat diet-induced inflammation leads to the hyperactivation of stress-sensitive Ser/Thr kinases, such as Jun kinase (JNK) and IkB kinase (IKKβ), which in turn inhibit the insulin receptor/ insulin receptor substrates 1 (IRS1) axis. The etiopathogenesis of insulin resistance involves a multitude of metabolic pathways; however, in recent years, all these metabolic pathways seem to be either directly or indirectly linked to increased inflammation. Indeed, chronic metabolic inflammation—called metaflammation—in multiple organs is involved in insulin resistance as well as in other metabolic diseases (reviewed in Hotamisligil [15]). A high conserved crosstalk has been well-established between immune and metabolic pathways. For example, the immune pathway, mediated by tumour necrosis factor (TNF), inhibits insulin signalling pathways through JNK activation. In this crosstalk, immune mediators, such as TNF, can act as metabolic hormones to adapt to nutritional status.

At the cellular level, endoplasmic reticulum (ER) stress [16,17,18,19], oxidative stress and mitochondrial dysfunction [1,3,20] have been proposed as mechanisms to explain the link between inflammation and insulin resistance [21]. In conditions of chronic overnutrition and positive body energy balance, both mitochondria (the powerhouse of the cell) and ER (the building factory of the cell) can experience stress due to the overflow of substrates and metabolic pressure. Mitochondria are functionally and structurally linked to ER, which in condition of chronic overnutrition undergoes stress, which activates the unfolded protein response (UPR) which in turn activates the principal inflammatory pathways that impair insulin action. Although the broader topic of immunometabolism or metaflammation will not be treated in detail in this review, this concept is very important for providing insight into the manipulation of immune responses to prevent or treat metabolic chronic disease [15]. For the aim of this review, it is important to underline that structurally and/or functionally impairment of intracellular organelles (namely ER and mitochondria) may serve as a crucial event in metabolic homeostasis and in the onset and the development of metaflammation [15].

Considering the suggested key role of inflammation/metaflammation in insulin resistance onset, nutrients and/or bioactive compounds with anti-inflammatory properties may be important in the prevention and therapy of insulin resistance. Among the nutrients, omega 3 polyunsaturated fatty acids (PUFA) have shown to have bioactive properties linked to their well-known anti-inflammatory effects [22,23,24]. Therefore, we reviewed the current knowledge on the regulatory effects of omega 3 PUFA on mitochondrial function, ER stress and inflammatory pathways in insulin resistance aetiology. We conducted a literature search which included studies in vitro and animal models as well as in human beings. In the first part of the review, we summarized the current hypothesis on the role played by mitochondrial and ER stress and inflammation in insulin resistance onset, whereas in the second part we focused on the modulatory role of omega 3 PUFA on these pathways. Understanding the mechanisms by which omega 3 PUFA modulate cellular metabolism and inflammatory pathways, may provide additional details to the potential impact of omega 3 PUFA on metabolic function and management of insulin resistance in humans.

## 2. Mechanisms Linking Mitochondria and Insulin Resistance

The complex nature of mitochondrial function has led to the development of a number of theories, sometimes with opposite views, describing the mechanisms linking mitochondria and insulin resistance. Association between mitochondria and insulin resistance have typically been focused on reductions of mitochondrial function in insulin resistant experimental models [25]. However, due to the results of several studies showing no mitochondrial function impairment in insulin resistant humans, the most accredited recent theory on mitochondrial involvement in the etiopathogenesis of insulin resistance focuses on reactive oxygen species (ROS) production [26,27].

### 2.1. Mitochondrial Dysfunction or ROS Production?

**Figure 1:**
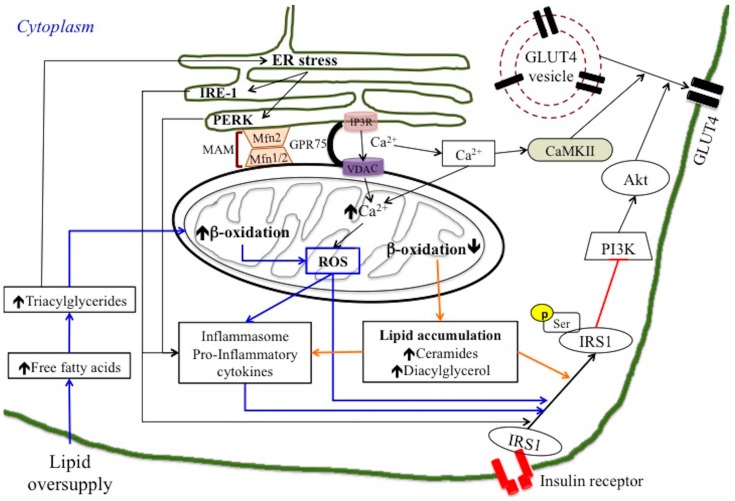
Overview of possible routes of lipid oversupply-induced insulin resistances associated with mitochondrial and endoplasmic reticulum (ER) stress. (Orange arrow) Mitochondrial dysfunction and lipotoxicity theory: lipid oversupply elicits reduction in mitochondrial β-oxidation that induces cytosolic ectopic accumulation of fatty acid metabolites, such as diacylglycerol and ceramides. These lipid metabolites impair insulin signalling pathways, directly or indirectly, through the activation of an inflammasome/pro-inflammatory pathway. (Blue arrow) ROS implication: lipid overload induces an increase in mitochondrial β-oxidation that stimulates ROS production. ROS can directly target protein involved in the glucose uptake process or activate the inflammasome, which initiates a series of inflammatory reactions in response to stress. ROS production may be also induced by excessive mitochondrial Ca^2+^ influx from ER. (Black arrow) ER stress induced by lipid oversupply directly actives inflammatory pathways and impairs insulin action. On the other hand, ER stress also trigger Ca^2+^ mobilization towards mitochondria through mitochondria-associated endoplasmic reticulum membrane (MAM), where Mfn2 play a regulatory role. The subsequent opening of the mitochondrial transition pore leads to activation of inflammasome and inflammatory pathways.

Initial theories suggested that impaired mitochondrial function results in compromised lipid β-oxidation, which in turn leads to ectopic lipid accumulation in peripheral tissues (lipotoxicity theory) (Figure 1) [20,25,28]. As previously discussed, the increase in lipid metabolites, such as ceramides and DAG, has been suggested to activate kinases involved in the impairment of insulin signalling, mainly at the level of the IRS1 [1,29]. In skeletal muscle, the impairment in insulin signalling pathways led to a decrease in GLUT4 exposition and a subsequent decrease in cellular glucose uptake (Figure 1). In this case, improvement of insulin sensitivity could be obtained by increasing lipid β-oxidation. In fact, this theory was supported by interventional studies where fatty acid oxidation rates have been increased with a resultant protection against insulin resistance [30].

Numerous animal and human studies confirmed the association between type 2 diabetes and reduced oxidative capacities [1,27]. Impairment of mitochondrial oxidative capacity was attributed to mitochondrial mass reduction in some studies [28] and/or reduced oxidative phosphorylation capacity per mitochondria in others [31,32]. However, the concept that mitochondrial dysfunction is a primary cause of insulin resistance is still being debated [33,34]. Indeed, increased lipid β-oxidation, rather than reduced β-oxidation, was reported in early stages of obesity and insulin resistance [35]. In addition, mitochondrial deficiency and impaired fat oxidation causes an increase, not a decrease, in insulin action [26]. These studies suggest that a deficiency of mitochondria in skeletal muscle does not play a causative role in insulin resistance (reviewed by Holloszy [26]).

An alternative mechanism to explain the connection between mitochondria and insulin resistance focused on ROS production (reviewed in [26,36]). In this case, insulin resistance is suggested to be caused by the production/emission of mitochondrial oxidants due to excess fuel within mitochondria in the absence of increased energy demand [37] (Figure 1). Indeed, lipid overload within the mitochondria results in the accumulation of partially oxidized acyl-carnitines, increased mitochondrial hydrogen peroxide (H_2_O_2_) emission, and a shift to a more oxidized intracellular redox environment. This oxidized redox environment may induce insulin resistance by directly targeting the protein involved in the glucose uptake process [3,38]. On the other hand, it has been suggested that the alteration in the cellular redox state towards oxidation could reduce the global serine/threonine phosphatase activity, which would result in increased activity of the serine/threonine stress sensitive kinases that inhibit the insulin signalling pathway, thereby inducing insulin resistance [1,3].

Although the primary role of skeletal muscle mitochondrial dysfunction in the pathogenesis of insulin resistance and type 2 diabetes is under debate [1,3], it is generally accepted that in this disease a mitochondrial defect occurs, possibly secondary to a fat intake increase. Moreover, association between mitochondrial dysfunction and insulin resistance was also observed in liver and adipose tissue. A clear link between reduced mitochondrial capacity and insulin resistance has been established in both visceral and subcutaneous adipose tissue [39]. Studies in animal models also showed associations between insulin resistance and hepatic mitochondrial dysfunction. Diabetic animals had reduced oxidative capacity in isolated hepatic liver mitochondria [40] and impaired hepatic lipid oxidation has been reported in conditions involving insulin resistance [41,42]. Moreover, reduction in absolute hepatic ATP concentrations and in ATP turnover were found in type 2 diabetes patients [43]. 

### 2.2. Mitochondrial Dynamic Behaviour: Role of Mitofusin in Insulin Resistance

In the last decade, research also focused on the role of mitochondrial dynamic behaviour, in terms of balance between fusion and fission processes, in insulin resistance development. Mitochondrial morphology is highly variable and it is maintained through a dynamic balance between fusion and fission processes [44]. These processes allow mitochondria to redistribute in a cell, exchange contents and repair damaged mitochondria. The fusion process is finely regulated by the mitochondrial fusion proteins—mitofusins 1 and 2 (Mfn1 and Mfn2)—located on the outer mitochondrial membrane, and the optic atrophy gene 1—OPA1—located on the inner mitochondrial membrane. Fission process is regulated by mitochondrial fission protein dynamin-related protein 1 (DRP1), a cytosolic protein recruited on outer mitochondrial membrane by fission protein 1 (Fis1) [45]. Mitochondrial dynamic behaviour has been suggested to play a key role in mitochondrial health, bioenergetics function, quality control, and cell viability. In the livers of insulin-resistant *db*/*db* mice, enhanced fission processes were associated with mitochondrial dysfunction. In addition, rats fed a high fat diet have lower Mfn2 gene expression than control rats, and this lower expression is accompanied by attenuated insulin signalling in the liver [46,47]. Overexpression of Mfn2 compensates for high fat diet-mediated disruption of insulin signalling [48]. Liver specific Mfn2 KO mice have higher levels of mitochondrial fragmentation and glucose intolerance and lower responses to insulin in the liver [49]. In addition, an association between increased mitochondrial fission and fat induced-insulin resistance has also been suggested in skeletal muscle [50]. Indeed, decreased skeletal muscle mitochondrial size and Mfn2 expression was found in obesity and type 2 diabetes [18,51,52]. In agreement, a positive correlation between Mfn2 expression and insulin sensitivity has been reported in skeletal muscle [53,54,55]. All these reports suggest that disruption of mitochondrial dynamics play a role in insulin resistance and type 2 diabetes with special emphasis on the role of Mfn2.

## 3. Mechanisms Linking ER Stress and Insulin Resistance

ER stress has been suggested to be an important contributor to insulin resistance [56]. At cellular level, increased levels of triglycerides are associated with the stress of the ER, which, by activating the UPR, may induce inflammatory response (Figure 1) [17,18,57,58]. In the condition in which the influx of the nascent unfolded polypeptides exceeds the processing capacity of the ER, UPR is activated leading to restoration of cell function. If the stressful signals are maintained for prolonged periods of time the ER stress lead to cell death. The UPR response is triggered by the activation of three main sensors of ER stress: inositol requiring kinase 1 (IRE1), activating transcription factor 6 (ATF6) and protein kinase-like ER kinase (PERK). IRE1 phosphorylates JNK, which in turn leads to impaired insulin signalling by inhibiting IRS1 (by phosphorylation at Ser307) and IkB kinase (IKK), which leads to the activation of NFkB and the inflammatory response. It is important to address that ER- and inflammatory-stress pathways are activated when the UPR or the ER-associated protein degradation (ERAD) of damaged or dispensable proteins via the ubiquitin-proteasome system (UPS), fail to restore ER function in response to cellular stress conditions.

ER stress has been shown to be associated with insulin resistance in different peripheral tissues. The first demonstration of the role of ER stress in insulin resistance came from studies by Ozcan et al. [16], which used cell culture and mouse models to show that obesity causes ER stress, which is itself a central feature of peripheral insulin resistance. Indeed, chronic lipid oversupply leads to an alteration of the ER membrane phospholipid composition and loss of membrane fluidity due to the accumulation of free cholesterol and saturated fatty acid-containing phospholipids in ER membrane. In skeletal muscle, this alteration of the ER membrane composition causes the inhibition of sarcoendoplasmic reticulum Ca^2+^-ATPase 2b (SERCA2b) activity, leading to ER Ca^2+^ depletion and protein misfolding [59]. Similarly, impaired ER Ca^2+^ content has been reported in hepatic ER stress in *ob*/*ob* mice [60]. In liver and adipose tissues, ER stress induced increase in JNK activity with subsequent impairment of insulin signalling. Chemical chaperones improved insulin sensitivity by blocking ER stress-mediated JNK activation [16]. As concern BAT, a very recent study highlighted the role of ERAD through the UPS as mechanisms by which brown adipocytes restore ER function and improve insulin sensitivity in animal models [61]. It showed that the ER-localized transcription factor nuclear factor erythroid 2-like 1 (Nrf1), a transcription factor embedded in ER membrane, is a fundamental regulator of the BAT adaptation to obesity-induced metabolic stress. Indeed, the stimulation of proteasomal activity by exogenously expressing Nrf1 in BAT improved systemic insulin sensitivity and glucose homeostasis in genetic and dietary obesity mouse model [61].

Notably, ER stress has been suggested to play a minor role in lipid-induced insulin resistance in skeletal muscle compared with the liver and adipose tissue [16,62]. Indeed, the initial study by group of Ozcan [16] suggested that ER stress is not induced in skeletal muscle in insulin-resistant mouse models. However, other studies demonstrated lipid induced ER stress in skeletal muscle. Indeed, palmitate exposure induced ER stress in human primary myotubes [63] and high-fat feeding activated the UPR in mouse skeletal muscle. Therefore, a lipotoxic state in skeletal muscle may induce ER stress, which may be potentially involved in skeletal muscle insulin resistance [64]. Nevertheless, ER stress does not seem to play a key role in muscle insulin resistance, because it has been reported that chemical chaperones which reduced ER stress, did not improve palmitate-induced alterations of insulin signalling in myotubes, [65]. To explain this finding, it is important to consider that palmitate was also reported to alter mitochondria and to induce oxidative stress in skeletal muscle [66,67]. It can be therefore suggested that mitochondrial dysfunction and/or oxidative stress rather than ER stress plays the key role in lipid-induced insulin resistance in skeletal muscle. Noteworthy, accumulated evidence suggests a potential interrelationship between alterations in mitochondria and ER, as mitochondrial dysfunction could participate in activation of the unfolded protein response, whereas ER stress could influence mitochondrial function [19]. Indeed, the traditional view of the ER and mitochondria as discreet intracellular organelles has been profoundly modified in recent years and interrelated roles have been suggested to contribute to insulin resistance onset [19,67].

## 4. ER-Mitochondria Interaction in Inflammation and Insulin Resistance

ER and mitochondria play their own distinct roles in cellular metabolism, but recent studies underlined the functional importance of the physically interaction between these two organelles [19,67,68,69,70]. The contact between mitochondria and ER is known as mitochondria associated ER membrane (MAM) and is responsible for efficient communication between these organelles exchanging calcium ions, lipids, and other metabolites to maintain cellular metabolism and integrity [71]. The physical interactions between both organelles depend on complementary membrane proteins, which tether the two organelles together at specific sites. For example, the inositol 1,4,5-triphosphate receptor (IP3R) on the ER interacts with the voltage-dependent anion channel (VDAC) of the outer mitochondrial membrane through the molecular chaperone glucose-regulated protein 75 (Grp75), allowing Ca^2+^ transfer from the ER to mitochondrial intermembrane space (Figure 1) [72,73]. Following this, the mitochondrial calcium uniporter (MCU), located in the mitochondrial inner membrane, imports calcium from the mitochondrial intermembrane space to the mitochondrial matrix. Recently, Mfn2 was discovered as a direct ER-mitochondria tether, regulating also the interactions and Ca^2+^ transfer between both organelles [74]. In addition to its localization to the mitochondrial outer membrane, Mfn2 that is crucial protein modulating mitochondrial tethering and fusion, also localizes to the ER membrane. Its localization to the ER membrane allows it to bind to Mfn1 or Mfn2 present on adjacent mitochondria, which promotes the formation of a bridge between the ER and mitochondria (Figure 1). Subsequently, Mfn2 deficiency leads to a reduction in mitochondrial calcium uptake rate [74]. Defects in MAM function and/or mitochondrial dysfunction may therefore lead to dysregulation of Ca^2+^ homeostasis which has been suggested to be involved in the pathogenesis of insulin insensitivity and type 2 diabetes [75,76].

It is well known that the intracellular level of Ca^2+^ ions in a normal human cell is regulated and maintained within a small range of concentration. In physiological conditions, an increase of the cytosolic level of Ca^2+^ ions initiates the activation of insulin signalling. For instance, in skeletal muscle and adipose tissue, cytosolic Ca^2+^ ions activate Ca^2+^/calmodulin-dependent protein kinase II (CaMKII), which allows translocation of glucose transporter 4 (Glut4) to the plasma membrane upon insulin stimulation (Figure 1). Mitochondria play a key role in the buffering of cytosolic Ca^2+^ ions due to their high capacity of Ca^2+^ uptake through the MCU complex and interaction with ER via the MAM structure [77]. In cellular stress conditions, the extreme accumulation of Ca^2+^ ions in the mitochondrial matrix elicited enhances ROS generation, which eventually leads to a collapse in mitochondrial function and apoptosis, via the opening of mitochondrial permeability transition pores (mPTPs) [78] and the activation of inflammatory pathways (Figure 1).

Therefore, it could be suggested that chronic overnutrition-induced ER stress [16] elicits extreme flux of Ca^2+^ ions in mitochondrial matrix which in turn causes oxidative stress and/or mitochondrial dysfunction inducing impairment of cellular response to insulin. Interestingly, increased oxidative stress and ER stress as well as activation of JNK was found in condition of MFN2 deficiency [48] and the specific loss of Mfn2 in hepatocytes was associated with hepatic insulin resistance [49]. In agreement, overexpression of Mfn2 improved diet-induced hepatic insulin resistance and chemical chaperones ameliorated glucose tolerance and insulin signalling in liver-specific Mfn2 KO mice [48]. These findings suggest a unique role of Mfn2 in the coordination between mitochondria and ER function as well as in the modulation of insulin signalling and glucose homeostasis.

In agreement, ER-mitochondria interactions are altered in liver of both *ob*/*ob* and diet-induced insulin-resistant mice [70]. Tubbs et al. demonstrated that MAM integrity is required for insulin signalling by performing experiments on cyclophilin D (CypD), a mitochondrial protein that interacts with the Ca^2+^-channelling complex at the MAM interface. They showed that genetic or pharmacological inhibition of CypD disrupted MAM integrity and altered insulin signalling in mouse and human primary hepatocytes. On the other hand, induction of MAM by overexpressing CypD enhanced insulin action in primary hepatocytes of diabetic mice [70]. More recently, Shinijo et al. showed that the disruption of MAM plays a significant role in palmitic acid-induced insulin resistance [79]. Palmitic acid treated HSPG2 cells showed a reduction of calcium flux from ER to mitochondria and a significant decrease in MAM contact area. It can be suggested that palmitic acid suppressed the mitochondria/ER functional interaction. In agreement, the overexpression of Mfn2 partially restored the MAM contact area and ameliorated the palmitic acid-induced insulin resistance, improving Ser473 phosphorylation of Akt [79]. This finding confirms the important role of Mfn2 in MAM integrity and functionality, and shed light on further possible interaction between ER and mitochondrial fusion/fission processes. In this context, it should be noted that MAM also plays a key role during the mitochondrial fission process by wrapping the damaged mitochondria with ER membrane. Moreover, mitochondrial fission has been shown to be connected to inflammation. Indeed, the bacterial component lipopolysaccharide (LPS) induces Drp1 translocation from the cytosol to mitochondria and promotes mitochondrial fission, accompanied by increased expression of genes encoding proinflammatory cytokines. By contrast, blocking Drp1 translocation to mitochondria or knocking down the Drp1 gene results in downregulation of proinflammatory cytokine gene expression in LPS-stimulated microglial cells [80]. This finding suggests a complex link among ER stress, mitochondrial dynamic behaviour and inflammatory process involving MAM interaction. As previously discussed, MAM provides a platform allowing exchange of biological molecules between the ER and mitochondria to maintain cellular homeostasis. However, abnormal exchange of these cellular metabolites due to excess nutrient intake, impairs mitochondrial health and promotes increased production of ROS and release of mitochondrial danger-associated molecular patterns into the cytosol. These molecules are recognized by inflammasome components, triggering increases in proinflammatory cytokine secretion to initiate inflammatory responses. Indeed, it is known that mitochondrial ROS activates the inflammasome, which is a multiprotein complex that initiates a series of inflammatory reactions in response to stress (Figure 1) [81,82]. Many of the signalling pathways activated by inflammatory response are serine/threonine kinases that can impair insulin signalling, such as JNK [83]. Inflammasome activation has been also shown to be induced by release of mitochondrial DNA as well as by increased lipid metabolites such as ceramides associated with impaired mitochondrial function (Figure 1) [84,85]. The exact role of mitochondrial control of inflammation in the aetiology of insulin resistance is not well established, but it provides another potential mechanism by which altered mitochondrial function could impact insulin action.

Collectively, these new findings suggest that research focusing on the complex interaction among mitochondrial function/dynamic behaviour, oxidative stress, ER stress and inflammatory pathways might shed light on novel strategy for prevention and therapy of insulin resistance. Therefore, studies on the modulatory role of nutrients and bioactive compounds on this complex interaction, rather than on the single pathway dysfunction, are needed to understand their potential use in insulin resistance prevention/therapy.

## 5. Omega 3 PUFA as Important Bioactive Lipids

Omega 3 PUFA have been suggested to be the most important bioactive lipids providing health benefits either through modification of tissue fatty acid composition or induction of cell signalling pathways (reviewed in Xu, 2015) [86]. Omega 3 PUFA include the essential α-linoleic acid (ALA) and longer-chain fatty acids, eicosapentaenoic (EPA) and docosahexaenoic (DHA), derived from marine organism. ALA is an essential fatty acid and it must be derived from dietary sources because it cannot be synthesized by humans due to a lack of the endogenous desaturase enzymes required for its production. In human tissues, ALA is then converted into the long-chain omega 3 PUFA, EPA or DHA, but the conversion pathways of ALA in EPA and DHA are very slow and therefore also EPA and DHA have to be ingested through diet and they can be considered essential fatty acids in normal conditions. The omega 3 PUFA are obtained predominately from fish [87] and in recent years from enriched food products such as milk and bread. Omega 3 PUFA beneficial health effects seem to be dependent on their *cis*-isomer configuration that is the predominant bioactive form. They are extremely flexible molecules due to the long tail of double bonds that grant them some unique chemical and physical properties. Omega 3 PUFA are required for the structure of cell membranes and help keep membranes flexible through their structural properties. Increased membrane fluidity helps maintain normal homeostasis and enhances cell-to-cell communication. Omega 3 PUFA may play their beneficial role on metabolic disorders prevention through their effects on cell membrane structure and/or modulating the expression of gene by regulating transcription factors related to energy supply and cell cycle [24,88]. In addition, it is well known that the main omega 3 PUFA positive effect is attributed to their ability to reduce inflammation [22,24], which is the hallmark of obesity and related metabolic disease. Indeed, established positive properties of omega 3 PUFA are their anti-inflammatory action and lowering effect on triglycerides [24,89], whereas controversy still exists on their effect on insulin action and glucose homeostasis.

In recent years, the role played by omega 3 PUFA in promoting health benefit has been the subject of an increasing number of studies [24,86]. Studies in rodents and humans have indicated that omega 3 PUFA potentially elicit effects which might be useful for reducing obesity and related metabolic disease, including enhanced fat oxidation and energy expenditure and reduced fat deposition [2,90]. Reducing body weight gain could be the first step to prevent or treat obesity-related disease, such as insulin resistance. Research has been carried out in cell culture, animal models, and human beings. An extensive review on current knowledge on potential role of omega-3 to improve insulin sensitivity has been recently published [24]. The review reported several studies supporting beneficial effects on insulin sensitivity in animal models. Human intervention studies fail to show benefit in type 2 diabetes and insulin resistant non-diabetic people, whereas observational studies in humans seems to be encouraging. 

In the context of this discussion on the potential impact of omega 3 PUFA on metabolic function in humans, in the present review we aimed to provide an up-to-date commentary on the potential mechanisms by which omega 3 PUFA may play beneficial effects on insulin sensitivity at the cellular level. As above discussed, insulin resistance is strictly linked to inflammation pathways development as well as to mitochondrial dysfunction, oxidative stress and ER stress. In the next paragraphs, we review the literature about how the well-known anti-inflammatory effect of omega 3 PUFA may also be linked to the amelioration of mitochondrial function and ER stress, contributing to counteracting insulin resistance onset.

## 6. Omega 3 PUFA Regulation of Mitochondrial Bioenergetics and Dynamic Behaviour

As previously discussed, decrease in mitochondrial lipid oxidation may lead to ectopic accumulation of lipids and their toxic intermediates (ceramides and DAG), which interfere with normal mitochondrial function and insulin signalling (lipotoxicity theory) [24,91,92].

**Figure 2:**
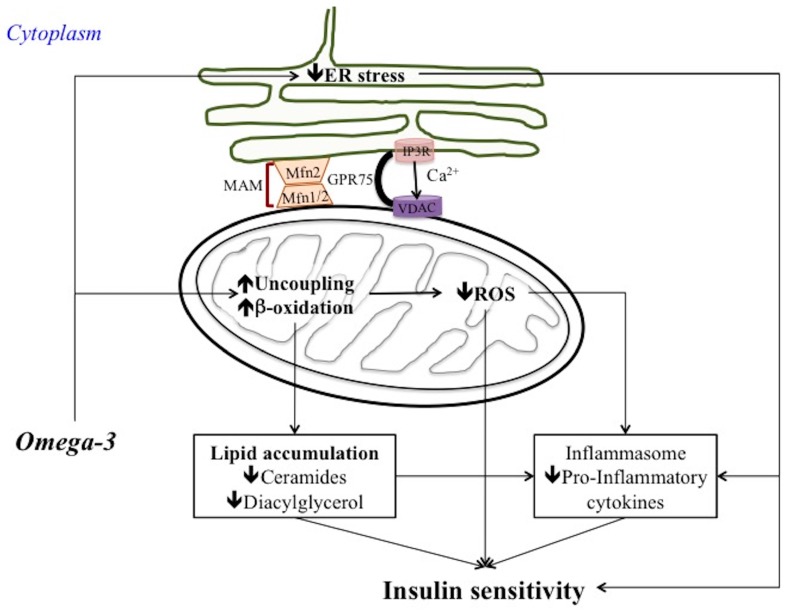
Proposal mechanism of omega 3 PUFA effects on insulin sensitivity. Omega-3 PUFA attenuate ER stress and increase mitochondrial fatty acid β-oxidation and mitochondrial uncoupling with subsequent decrease in lipid accumulation and ROS production. Therefore, inflammasome/inflammatory processes are downregulated improving insulin sensitivity. Omega 3 PUFA have also positive effects on Mfn2 involved in maintenance of mitochondrial dynamics homeostasis and MAM integrity. Mitochondrial fusion phenotype and MAM integrity contribute to maintain insulin sensitivity under condition of cellular stress.

A number of studies suggested that omega 3 PUFA can prevent or reverse impairments in mitochondrial function or content in skeletal muscle. Indeed, fish oil elicited an increase in the expression of the transcriptional factors of mitochondrial biogenesis, including peroxisome proliferator-activated receptor gamma coactivator 1 α (PGC1a) and nuclear respiratory factor-1 (NRF1), in the skeletal muscle of mice fed a high fat diet (60% fat) with fish oil (3.4% kcal from *n*-3 PUFAs) for 10 weeks [93]. Moreover, an increased dietary intake of long chain omega 3 PUFA (high fat diet (200 g fat/kg) containing menhaden (fish) oil) has been shown to induce an increase in mitochondrial carnitine palmitoyl transferase 1 (CPT-1) in rat hearts and skeletal muscle [94]. CPT-I is the main control point for β-oxidation because it facilitates the transfer of acyl groups into the mitochondria, and its expression is regulated by peroxisome proliferator-activated receptors (PPARs) and 5′-AMP-activated protein kinase (AMPK). It has been shown that the observed increase in mitochondrial CPT-1 expression and fatty acid oxidation was due to the activation of AMPK by EPA (200 mumol/L for 24 h) in both primary cultured rat adipocytes [95] and the skeletal muscle of high fat fed rats supplemented with 10% *v*/*w* omega 3 PUFA (as fish oil) for 6 weeks [96]. The increased fatty acid utilization is likely to contribute to decrease ectopic lipid accumulation and lipotoxicity playing an important role in counteracting insulin resistance onset (Figure 2). Noteworthy, in the liver the increase in fatty acid utilization induced by omega 3 PUFA has been found to be also associated with increased mitochondrial uncoupling which in turn counteracts ROS production and subsequent inflammatory pathways and insulin signalling impairment [47] (Figure 2).

In addition to their effects on the skeletal muscle, heart, and liver, omega-3 fatty acids increased fat oxidative, and decreased fat synthesis, capacities in visceral (epidydimal) fat depots in C57BL/6J mice fed a high-fat diet supplemented with EPA/DHA concentrate (6% EPA, 51% DHA) [97]. The increased fat oxidation in visceral fats resulted from upregulated levels of CPT-1, as well as of PGC-1α and NRF-1 that regulate mitochondria biogenesis. A reduction has also been found in the mRNA expression of stearoyl-CoA desaturase—the key enzyme in the lipogenic pathway—in epidydimal fat. Taken together, the findings of this study [97] indicate that omega 3 PUFA increase the expression of genes in visceral fat which favour fatty acid oxidation and reduce fatty acid synthesis.

Another mechanism proposed for the adipose tissue effects (decreased fat deposition) of omega 3 PUFA is by way of increased expression of uncoupling protein 3 (UCP-3) mRNA in skeletal muscle, coupled with increased expression of peroxisomal acyl-CoA oxidase (PACO) in liver, heart, and skeletal muscle [98]. In this study, male Fisher 344 rats were fed for 6 weeks a diet containing 40% of its energy fat derived by fish oil. UCP3 and PACO contributes to dissipate energy from lipid substrates in heat production counteracting fat deposition. Indeed, UCP3 induce mitochondrial proton leakage leading to reduction in the efficiency of ATP production from oxidative phosphorylation and to increase in heat production. On the other hand, the increased PACO expression indicates an increase in peroxisomal fatty acid oxidation that is a much less efficient pathway for energy production than mitochondrial β-oxidation, with 30% less ATP and 30–40% more heat production than mitochondrial oxidation. Therefore, the combination of an increased UCP3 and peroxisomal acyl–CoA oxidase expression results in a reduction in overall metabolic efficiency, leading to an increased loss of energy in the form of heat and less storage of energy in the form of fat. Given that fat accumulation both in WAT and in ectopic tissues is associated with the onset of insulin resistance, the increase in fatty acid utilization and the less efficient pathway of energy production through mitochondrial uncoupling and/or increased peroxisomal fatty acid oxidation leading to less lipid accumulation may explain the beneficial effect of omega 3 PUFA on insulin sensitivity.

Another aspect to be considered when analysing the beneficial effects of omega 3 PUFA on insulin resistance is the impact on mitochondrial dynamic behaviour (reviewed in Putti et al. [27]). Indeed, several studies underlined the potential role of mitochondrial morphology and dynamic behaviour in determining mitochondrial dysfunction and insulin resistance onset, as previously discussed. We recently showed that the replacement of lard with fish oil (40% fat J/J). Over 6 weeks, high fat feeding positively affected mitochondrial dynamic behaviour in association with an improvement of insulin resistance in both skeletal muscle and liver in Wistar rats [47,99]. In skeletal muscle, a high fish oil diet, compared to a high lard diet, ameliorated systemic insulin sensitivity and elicited IRS1 and pIRS (Tyr632) immune-reactivity, similar to a control diet [99]. This beneficial effect of fish oil feeding on skeletal muscle insulin resistance development was associated with changes in mitochondrial dynamic behaviour. Indeed, a greater number of immunoreactive fibres in Mfn2 and OPA1 proteins was observed in the skeletal muscle of high-fish oil fed rats compared to high-lard fed ones. This observation, together with the weaker immunostaining for DRP1 and Fis 1 observed in high-fish oil fed versus high lard fed rats, suggested a shift towards a fusion process induced by high fish oil feeding. In agreement, skeletal muscle electron microscopy observations suggested a prominent presence of fusion events in high-fish oil diet fed rats [99]. This finding is in accordance with an in vitro cultured L6 myocytes study, which showed that DHA (25 μM for 4 h) induced the downregulation of fission genes DRP1 and Fis1 associated with a higher proportion of large and elongated mitochondria [100]. Mitochondrial dynamics has been suggested to be associated with the inflammatory processes involved in insulin resistance development. Indeed, inflammatory parameters may play a regulatory role on MFN2. It has been shown that TNF-α inhibits Mfn2 gene expression in cells in culture [101] and, in addition, dietary saturated fatty acids, known to have proinflammatory effects, reduced Mfn2 expression and induced fission phenotype in skeletal muscle [99]. On the other hand, anti-inflammatory effect of dietary omega 3 PUFAs was associated with a tendency to mitochondrial fusion without reduction in skeletal muscle Mfn2 content [99].

In agreement with the pro-fusion effect of omega 3 PUFA dietary fat on skeletal muscle mitochondria, the positive effect of omega 3 PUFA on liver inflammation and insulin resistance was also associated with improved mitochondrial function and shift towards fusion processes. Indeed, differently from saturated fatty acids, omega 3 PUFA improved mitochondrial function, reduced ROS production, and promoted hepatic mitochondrial fusion both in vitro and in vivo. In an in vitro steatotic hepatocyte model created using HepG2 cells, both EPA and DHA (50 μmol/L for 1 h) induced a decrease in oxidative stress associated with increased Mfn2 expression and ATP levels [102]. These omega 3 PUFA effects were not elicited in Mfn2-depleted steatotic hepatocytes [102], confirming an important role for Mfn2 in the mechanism of the regulation of mitochondrial function by omega 3 PUFA (Figure 2).

In agreement with these data obtained in steatotic hepatocytes in vitro, replacement of lard with fish oil in high fat diet (40% fat J/J for 6 weeks) in male Wistar rats prevented hepatic steatosis and insulin resistance by reducing ROS production, promoting fusion processes and ameliorating mitochondrial function [47]. Indeed, high fish oil diet did not elicit hepatic insulin resistance as shown by the determination of insulin-stimulated AKT phosphorylation (at Ser473), but it induced a lower degree of hepatic steatosis compared to the high lard diet, possibly through an ameliorated mitochondrial utilization of lipid substrates mediated by PPARα and associated with shift towards fusion phenotype. In the livers of high fish oil diet fed rats, it was also found an increased expression of uncoupling protein 2 and mild mitochondrial uncoupling which in turn might contribute to increase fatty acid utilization and reduce ROS production. These changes in mitochondrial function induced by high fish oil diet were associated with a shift towards fusion. An increase in the number of tubular mitochondria was indeed observed by electron microscopy in the liver of high-fish oil fed rats compared to high-lard fed ones, in agreement with an increase in fusion protein content and a decrease in fission ones [47]. In this way, high fish oil diet counteracted hepatocyte damage and insulin resistance induced by long-term high fat feeding [47]. The shift toward fusion may be an adaptive mechanism by which functional mitochondria complement dysfunctional mitochondria counteracting cellular stress induced by chronic high fat diet, and beneficial effect of omega 3 PUFA may be also due to the maintenance of fusion processes.

The mechanism underlying fish oil/omega 3 PUFA mitochondrial fusion stimulation may involve changes in lipid composition of mitochondrial membranes and/or receptor-mediated signalling. Further studies are needed to elucidate this mechanism as well as the mechanism linking mitochondrial fusion to amelioration of energy cellular metabolism. Moreover, complex interaction between mitochondrial function and dynamics and insulin resistance have been proposed. Therefore, it is auspicious that future studies, which aim to determine how effective targeting mitochondria could be for the treatment of insulin resistance by bioactive nutrients such as omega 3 PUFA, consider mechanisms involved in both mitochondrial function and dynamic behaviour. Given the role played by Mfn2 in ER-mitochondria structural and functional interaction, the regulatory role of omega 3 PUFA on this complex interaction need to be discussed.

## 7. Omega 3 PUFA Regulation of ER Stress, MAM and Inflammatory Process

### 7.1. Omega 3 PUFA Attenuate ER Stress

Different fatty acids seem to have different effect on ER stress and subsequent inflammatory process. It has been shown in vitro that saturated fatty acids (FA) (such as palmitic acid) can induced ER stress and apoptosis leading to inflammation and/or degeneration [103,104,105]. Study in vitro in primary rat hepatocytes showed that PUFA, such as alpha-linolenic acid (150 or 250 μmol/L for 16 h) protects against ER stress-mediated apoptosis of stearic acid lipotoxicity [106] and it has been suggested that may provide a useful strategy to counteract the lipotoxicity induced by dietary palmitic acid as well as by nutrient overload associated with obesity and insulin resistance [107].

A recent study analysed whether fish oil supplementation reduces ER stress and ameliorates insulin resistance in high fat diet-induced obese mice [108]. It showed that fish oil-rich high fat diet (60% fat derived calories, for 12 weeks) improved glucose tolerance and insulin sensitivity in high-fat diet-induced obese mice. This improved insulin sensitivity was associated with reduced adipose tissue dysfunction and inhibition in high-fat feeding-induced ER stress. Noteworthy, fish oil supplementation also induced an increase in phosphorylated AMP-activated protein kinase (AMPK) expression in WAT [108]. In agreement, increased AMPK phosphorylation and suppressed palmitic acid-triggered ER stress was observed in differentiated 3T3-L1 adipocytes treated with DHA (150 μM for 24 h). Therefore, omega 3 PUFA suppression of ER stress in adipocytes has been suggested to be due to AMPK activation. This hypothesis was confirmed by using AMPK inhibitor (compound C) which was able to block the effects of DHA to inhibit palmitic acid-induced ER stress. Fish oil has also been shown to decrease ER stress and reduce the percentage of apoptotic cell death in the pancreatic islets preventing β-cell dysfunction in obese KK mice with type 2 diabetes fed a diet with 10% energy fish oil for 8 weeks [109]. Resolvins, a novel family derived from EPA and DHA, have anti-inflammatory and insulin sensitizing properties and it has been suggested that they play a role in the amelioration of obesity-related metabolic dysfunctions. Indeed, resolvin D1 (10 nM) attenuated ER stress-induced apoptosis and decreased caspase 3 activity in HepG2 cells, preventing lipid accumulation and insulin resistance [110]. Collectively, beneficial effects of omega 3 PUFA on insulin sensitivity can be also due to their effect on reducing ER stress and subsequent inflammatory pathways linked to insulin resistance onset (Figure 2).

### 7.2. Omega 3 PUFA Inhibits Inflammasome Activation: Role of MAM

Omega 3 PUFA have been recently proposed as modulators of nucleotide-binding and oligomerization domain-like receptor, leucine-rich repeat and pyrin domain-containing 3 (NLRP3) inflammasome in obesity and obesity-related metabolic diseases, such as insulin resistance [111], and it has been suggested a putative involvement of mitochondria in this modulation [112]. Inflammasomes are multiprotein complexes that mediate the first line of the immune response defence through the activation of caspase-1 and the induction of IL-1β and IL-18 secretion under conditions of exposure to pathogens and host danger signals [113]. The NLRP3 inflammasome is the best-characterized inflammasome and is activated by a large variety of signals, including pathogen-associated molecular patterns and danger-associated molecular patterns [113]. It has been suggested that NLRP3 generally senses disturbance in cellular homeostasis, such as changes in redox status or ER stress. However, the precise mechanism of NLRP3 activation is not fully understood. An essential role of mitochondria in NLRP3 inflammasome activation has been suggested [114]. NLRP3 stimuli seem to induce a re-localization of NLRP3 from ER to MAM, where it forms a functional inflammasome with caspase-1 [81]. NLRP3 inflammasome activation may be promoted by microtubule- driven spatial arrangement of mitochondria [115] as well as by mitochondrial damage and increased mitochondrial ROS concentrations [81]. Moreover, NLRP3 may be activated directly by oxidized mitochondrial DNA [84] and phospholipid cardiolipin [116], which is usually localized in the inner mitochondrial membrane and is externalized upon mitochondrial damage [117]. Finally, it has been postulated that Ca^2+^ signalling induced by many stimuli could trigger mitochondrial destabilization which in turn activate the NLRP3 inflammasome [118]. Many NLRP3 triggers induce Ca^2+^ mobilization from ER, which is closely coordinated with mitochondrial Ca^2+^ uptake. Excessive mitochondrial Ca^2+^ influx can lead to mitochondrial damage and ROS production. The subsequent opening of the mPTPs leads to activation of NLRP3 inflammasome [119]. This model suggests that ER-mitochondria juxtaposition through MAM and the MCU are necessary for NLRP3 inflammasome activation. Therefore, Ca^2+^ fluxes may be the intermediate mechanism linking mitochondrial dysfunction, ER stress and inflammation pathways in insulin resistance development and omega 3 PUFA may regulate this mechanism (Figure 1 and Figure 2). Indeed, the NLRP3 inflammasome can be influenced by various metabolites, including fatty acids. The modulatory role of fatty acids on NLRP3 inflammasome-mediated inflammation in different metabolic tissues has been recently reviewed by Ralston et al. [120]. Saturated fatty acids induce NLRP3 inflammation activation, whereas monounsaturated and polyunsaturated fatty acids, especially omega-3 fatty acids, inhibit NLRP3 activity. Therefore, the NLRP3 inflammasome-mediated inflammation play a key role in the relationship between fatty acids and metabolic diseases such as insulin resistance. It has been shown that NLRP3 inflammasome can sense ceramides generated from saturated fatty acids under condition of lipid surplus and contribute to obesity-induced inflammation and insulin resistance [85]. A systematic review has recently been conducted by Rheinheimer et al. [21] to summarize the results of studies evaluating the association between the NLRP3 inflammasome and obesity and insulin resistance [21]. They concluded that overall human studies indicate that obesity and insulin resistance are associated with increased NLRP3 expression in adipose tissue and studies in obese mice corroborate this association. In addition, Yan et al. investigated whether the omega 3 PUFA exert their anti-inflammatory activity through the inhibition of inflammasome activity [111]. They showed that stimulation of bone-marrow-derived macrophages with omega 3 PUFA (DHA 20 μM), abolished NLRP3 inflammasome activation and inhibited subsequent caspase-1 activation and IL-1β secretion. In addition, they suggested that G protein-coupled receptor 120 (GPR120) and GPR40 were involved in inflammasome inhibition induced by omega 3. Noteworthy, omega 3 PUFA supplementation (100 mg/kg, twice a week for 10 weeks) also prevented NLRP3 inflammasome-dependent inflammation and metabolic disorder in a high-fat-diet-induced type 2 diabetes mouse model, revealing a mechanism through which omega 3 PUFA repress inflammation and prevent inflammation-driven diseases, such as insulin resistance [111]. More research will be needed to decipher the molecular and cellular mechanism leading to inhibition of NLRP3 by omega 3 PUFA. In particular, it would be interesting to test in this context the effect of omega 3 PUFAs on mitochondrial cardiolipin and/or Ca^2+^ flux between ER and mitochondria to elucidate whether omega 3 PUFA reverse or inhibit these mechanisms suggested to be involved in induction of NLRP3 inflammasome and insulin resistance onset. In addition, given that Mfn2 play an important role in both ER-mitochondria connection and in insulin resistance prevention, the link between Mfn2 and Ca^2+^ flux in mitochondria should be further investigate in omega 3 PUFA beneficial effect involving inflammasome inactivation. Indeed, considering that the NLRP3 inflammasome has been associated with insulin resistance development, these kinds of research studies will be useful to shed light on the potential clinical use of omega 3 PUFA both in prevention and therapy of insulin resistance.

### 7.3. Omega 3 PUFA and Metaflammation: Few Considerations

A very recent review focused on the importance of omega 3 PUFA in improving immunometabolism in WAT of obese mice by inducing “healthy adipocytes” phenotype [121]. The definition “healthy adipocyte” phenotype is referred to small adipocyte with high capacity for mitochondrial oxidative phosphorylation and resistant to ER stress and related inflammatory and insulin-resistance response. It should be noted that, in accordance with the concepts so far discussed, optimal mitochondrial function and ER homeostasis are strictly linked in the definition of “healthy adipocytes” and in insulin resistance prevention. It has been suggested that the improvement in WAT metaflammation by omega 3 fatty acids may be associated with the prevention of ectopic fat accumulation and lipotoxic impairment of the insulin pathway in extra adipose tissue, such as in the liver and skeletal muscle. These omega 3 effects on metaflammation, starting in adipose tissue and extending to metabolic peripheral tissues, explain the underlined cellular mechanisms of beneficial effect in overall systemic insulin resistance [17,18,121].

Taking into account the above considerations, it should be noted that efficient storage of omega 3 fatty acids seems to be a prerequisite for a healthy function in both adipose tissue and metabolic peripheral tissues. In this context, it should be underlined that lipoprotein lipase (LPL) has been shown to be an important regulator of PUFA delivery to peripheral tissue from triglyceride-rich lipoproteins [122]. LPL is a rate-limiting enzyme that catalyses hydrolysis of the triglyceride core of circulating chilomicrones, low-density lipoproteins and very low-density lipoproteins. Adipocyte-specific LPL knockout (aLKO) mice displayed a decrease in essential dietary fatty acids in BAT and WAT, confirming the important role of LPL in PUFA delivery to peripheral tissues [122]. Exploring this regulatory role of LPL in PUFA delivery may open new perspectives in the prevention and therapy of insulin resistance.

## 8. Conclusions

Omega 3 PUFA are bioactive lipids playing beneficial effects on human health due to their anti-inflammatory proprieties. Insulin resistance is strictly linked to inflammatory pathways which in turn is associated with ER stress, ROS production and mitochondrial function/dynamic behaviour impairment. Indeed, lipid oversupply from chronic overfeeding negatively affects the function of cellular organelles such as ER and mitochondria, that are no longer considered discreet intracellular organelles but sharing interrelated roles in insulin resistance onset, as confirmed by the fact that MAM integrity is required for insulin signalling. Omega 3 PUFA, differently from saturated fatty acids, stimulate mitochondrial function and fusion processes reducing ROS production due also to increase in mitochondrial uncoupling. In addition, omega 3 PUFA have been proposed to attenuate ER stress and subsequent disruption of Ca^2+^ homoeostasis as well as induction of inflammasome/inflammatory pathways. 

Given the unique role played by Mfn2 as ER-mitochondria bridge, the positive modulatory effects of omega 3 PUFA on Mfn2 may explain the induction of fusion processes linked to amelioration of mitochondrial function as well as to the maintenance of MAM integrity, both necessary for insulin sensitivity. Further studies are necessary to further clarify the specific role of mitochondria and ER in the bioactive effects of PUFA as well as to identify the cause-effect relationship among mitochondrial dysfunction, dynamic behaviour and ER/MAM dysregulation, and inflammatory processes. These kinds of studies considering the complex interaction between organelles stress and inflammatory process, rather than the single pathway dysfunction, in the wide frame of the concept of metaflammation, may be useful to clarify the mechanisms underlying the potential use of omega 3 PUFA as bioactive compounds to prevent or treat insulin resistance.
